# Noninvasive Imaging Methods to Improve the Diagnosis of Oral Carcinoma and Its Precursors: State of the Art and Proposal of a Three-Step Diagnostic Process

**DOI:** 10.3390/cancers13122864

**Published:** 2021-06-08

**Authors:** Antonio Romano, Dario Di Stasio, Massimo Petruzzi, Fausto Fiori, Carlo Lajolo, Andrea Santarelli, Alberta Lucchese, Rosario Serpico, Maria Contaldo

**Affiliations:** 1 Multidisciplinary Department of Medical-Surgical and Dental Specialties, University of Campania Luigi Vanvitelli, Via Luigi de Crecchio, 6, 80138 Naples, Italy; antonio.romano4@unicampania.it (A.R.); dario.distasio@unicampania.it (D.D.S.); fausto.fiori@outlook.com (F.F.); alberta.lucchese@unicampania.it (A.L.); rosario.serpico@unicampania.it (R.S.); 2 Interdisciplinary Department of Medicine, University of Bari “Aldo Moro”, Piazza Giulio Cesare 11, 70124 Bari, Italy; massimo.petruzzi@uniba.it; 3 Head and Neck Department, Fondazione Policlinico Universitario A. Gemelli–IRCCS, School of Dentistry, Università Cattolica del Sacro Cuore, Largo A. Gemelli, 8, 00168 Rome, Italy; Carlo.Lajolo@unicatt.it; 4 Department of Clinical Specialist and Dental Sciences, Marche Polytechnic University, Via Tronto 10, 60126 Ancona, Italy; andrea.santarelli@staff.univpm.it

**Keywords:** noninvasive diagnoses, OSCC, diagnosis, toluidine blue, Lugol’s iodine, tissue autofluorescence, high-frequency ultrasounds, optical coherence tomography, narrow-band imaging, virtual chromoendoscopy with magnification, fluorescent confocal microscopy, reflectance confocal microscopy

## Abstract

**Simple Summary:**

Oral squamous cell carcinoma (OSCC) accounts for 90–95% of malignant tumors of the lip and oral cavity and is associated with high mortality in the advanced stages. Early diagnosis is a challenge for oral pathologists and dentists, due to the ambiguous appearance of early OSCC, which is often misdiagnosed, mistreated, and associated with diagnostic delay. The gold standards for OSCC diagnosis are biopsy and histopathological assessment, but these procedures are invasive and time-consuming. Adjunctive noninvasive techniques allow the definition of the malignant features of a suspicious lesion in real time and noninvasively, thus improving the diagnostic procedure. The present review aimed to focus on some of the main promising noninvasive imaging techniques, to highlight their perspective adoption in a three-step diagnosis, which is idealistically faster and better, as well as enables the patient’s compliance.

**Abstract:**

Oral squamous cell carcinoma (OSCC) is the most prevalent form of cancer of lips and oral cavity, and its diagnostic delay, caused by misdiagnosis at the early stages, is responsible for high mortality ratios. Biopsy and histopathological assessment are the gold standards for OSCC diagnosis, but they are time-consuming, invasive, and do not always enable the patient’s compliance, mainly in cases of follow-up with the need for more biopsies. The use of adjunctive noninvasive imaging techniques improves the diagnostic approach, making it faster and better accepted by patients. The present review aims to focus on the most consolidated diagnostic techniques, such as vital staining and tissue autofluorescence, and to report the potential role of some of the most promising innovative techniques, such as narrow-band imaging, high-frequency ultrasounds, optical coherence tomography, and in vivo confocal microscopy. According to their contribution to OSCC diagnosis, an ideal three-step diagnostic procedure is proposed, to make the diagnostic path faster, better, and more accurate.

## 1. Introduction

Oral squamous cell carcinoma (OSCC) is a keratinocyte-derived malignancy affecting the oral mucosae of lips and oral cavity, accounting for 90–95% of malignant tumors of this anatomic region [1], while the remaining 5–10% is made up by rarer malignancies [2,3,4]. In 2020, the OSCC 5 year prevalence, annual incidence, and annual mortality worldwide, for both sexes and all ages, were 959,248, 377,713, and 177,757, respectively. To date, OSCC is the 11th most common malignancies in males, and the 16th in females (female/male ratio: 1:2) and is the seventh malignancy responsible for death in males between 30 and 70 years of age and the 14th for women of all ages [1].

Alcohol and/or tobacco consumption (whether smoked or not) [5], betel quid chewing [6], micronutrient deficiencies [7], chronic traumatism [8], and viruses [9,10,11] are classical and well-known OSCC risk factors. Furthermore, in recent years, the study of the oral microbiome [12] has brought to light the role of poor oral hygiene and oral dysbiosis [13,14] in the mechanisms responsible for aggravating and/or triggering both oral and extraoral neoplasms and diseases [15,16,17], due to the carcinogenic potential of various fungi and bacteria [18,19], through the perpetuation of chronic inflammation, the production of toxic metabolites, and directly carcinogenic substances, generally favoring oxidative stress and creating the ideal microenvironment for tumor growth [20,21,22,23,24].

OSCC often arises from precursor lesions and conditions with an increased risk of malignancy, recently grouped under the name of “oral potentially malignant diseases” (OPMDs) [25].

Delays in the diagnosis of OSCC are responsible for high mortality and morbidity, mainly related to the lymph nodal and distant metastases that frequently occur in late-stage OSCCs, which are related to shorter overall survival and poor quality of life of “survivors of advanced OSCC”, as a consequence of massive but necessary neck dissections and mutilating eradications of the primary tumor [26,27].

Despite easy access to the oral cavity, the varied clinical presentations of OSCCs are not always indicative of cancer and may mimic other oral lesions [28], which, when misdiagnosed or mistreated, mainly in the early stages [29], promote tumor growth and its invasiveness and metastasis [30,31,32].

In addition to prevention and elimination of risk factors, early diagnosis is the most powerful weapon to reduce OSCC mortality or the consequences deriving from its treatment in advanced stages. To date, the diagnostic procedure for diagnosing OSCC is time-consuming and invasive, since it must consider the multistep processes, always culminating in biopsy and histopathological assessment as gold standards [33]. Furthermore, while the malignant nature of those lesions in the advanced stages is easily identified, for the diagnostic recognition of early cancers and precancerous lesions, clinical inspection and palpation are by no means sufficient on their own, particularly for those lesions which are multifocal, doubtful, and large, thus requiring a proper presurgical mapping or multiple biopsies to establish the correct diagnosis. Although indispensable, biopsy and conventional histopathological assessment are time-consuming, whereas surgery may be associated with adverse reactions or postoperative complications, which do not always enable the patient’s compliance, mostly in cases with a need for multiple or repeated biopsies to follow up neoplastic evolution of OPMDs [34].

All these reasons lead oral pathologists and oncologists to constantly research and apply novel noninvasive imaging techniques, able to add information during the clinical examination to shorten the time for biopsy and guide it by selectively identifying and targeting only highly suspicious lesions at their most representative sites [35].

The present review aims to describe the methodology, basics principles, limitations, and future perspectives of a series of noninvasive imaging techniques, applied to oral cancer detection. In detail, on the basis of the direct experiences of the authors, the present review discusses the following: vital and virtual staining, tissue autofluorescence, narrow-band imaging, optical coherence tomography, high-frequency ultrasonography, and in vivo confocal microscopy.

## 2. Vital Staining: Toluidine Blue and Lugol’s Iodine

The vital dyes are consolidated auxiliary tools used in vivo, directly on the oral lesion, to evidence suspicious lesions and/or to better define their margins and extent. These dyes are nontoxic substances capable of penetrating living cells and binding to specific biological structures. In clinical practice, the most commonly used are toluidine blue (TB) and Lugol’s iodine (LI).

**Figure 1:**
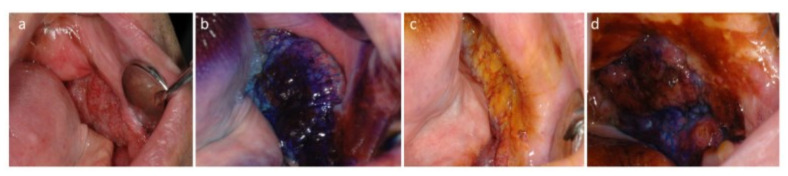
OSCC of the edentulous mucosa: (**a**) clinical presentation; (**b**) TB positivity (royal blue); (**c**) LI scarcely dyes the lesion, and brown-orange staining is limited to the periphery of the lesion; (**d**) TB and LI staining empower the identification of neoplastic areas (royal blue) and benign mucosa (brown-orange).

On the basis of its acidophilic properties, the metachromatic dye TB selectively stains tissues rich in nucleic acids [36]. Hence, neoplastic/highly dysplastic lesions, whose cells have a high content of DNA and RNA, clinically appear stained in royal blue (TB-positive) [37], while the healthy and nondysplastic/non-neoplastic tissues appear pale blue or do not capture the dye at all (Figure 1b) (TB-negative). To perform a correct TB staining procedure, the patient is asked first to rinse the mouth with water to remove plaque and debris; then, after applying a 1% aqueous solution of acetic acid for 1 min to make the cells more receptive, 1% TB stain is applied onto the suspicious lesion. After another minute, the patient rinses, and the excess dye is removed with acetic acid. TB is internalized in cancer/dysplastic cells, whose tissue appears dark blue (royal blue) [38].

Conversely, Lugol’s iodine (LI), also known as Lugol’s solution, marks the healthy tissues. LI is a solution generally consisting of 5 g of elemental iodine and 10 g of potassium iodide in 85 mL of distilled water with a total iodine content of 150 mg/mL [39]. The principle of iodine staining is that iodine reacts with iodine-starch reactions with cytoplasmic glycogen of the cells, clinically visualized by a color change (brown-orange). According to the fact that the glycogen content of cells is inversely proportional to the degree of keratinization, the loss of cellular differentiation and the enhanced glycolysis in cancer cells do not promote the iodine–starch reaction. Therefore, during mucosal examination under Lugol’s staining, normal mucosa is brown or orange due to its high content in glycogen, while dysplastic/neoplastic tissues do not stain, and they appear pale yellow compared to the surrounding tissue [39] (Figure 1c). LI and TB can also be used in association (Figure 1d), to strengthen the evidence for the dysplastic/neoplastic vs. healthy area within and around a mass (Figure 1d).

Epstein et al. compared the sensitivity and specificity of TB and LI alone and in association, revealing the sensitivity and specificity of 0.925 and 0.632 in TB and of 0.875 and 0.842 in LI, respectively. When associated, the two dyes showed a sensitivity of 0.850 and a specificity of 0.895, concluding that Lugol’s iodine has less sensitivity in identifying oral dysplasia and cancers but greater specificity, compared with TB [40].

## 3. Virtual Staining: Tissue Autofluorescence

As a sort of “virtual staining”, tissue autofluorescence (AF) is based on the detection of the proper fluorescence emitted by tissues irradiated with a specific wavelength of light. In detail, “fluorescence is the property of some intrinsic molecules called fluorophores to absorb light at a particular wavelength and to re-emit it at a longer wavelength” [41].

The light used in autofluorescence imaging, is blue light, at the wavelength of 400–460 nm, which, upon hitting the tissues, stimulates the emission of green fluorescence at the wavelength of 500–520 nm from endogenous fluorophores as keratin, collagen, elastin, and NADH; conversely, hemoglobin, porphyrins, and melanin tend to absorb the blue incident light, thus reducing tissue autofluorescence [38]. The intensity of the fluorescence decreases with the progress of the dysplasia; progressing from mild dysplasia to carcinoma, the fluorescence decreases, until it disappears in the case of neoplastic lesions, which, like a black hole, absorb all the incident light, thus appearing negative to the fluorescence and completely dark [42]. In addition toTB, AF is, therefore, useful to discriminate malignant lesions, to identify early tumors, carcinomas in situ, and tumor recurrences, even before pathognomonic clinical manifestations occur [43]. The causes responsible for the loss of fluorescence in malignant lesions are linked to various biochemical and architectural changes, which alter the correct backscattering of the fluorescent light [38,42].

The light source to stimulate tissue autofluorescence is any blue light at the wavelength of 400–460 nm, but specific filters are necessary to detect the green fluorescent light emitted by the tissues [44].

The examination must be performed in a dark room, and the emitted fluorescence can be recorded with digital cameras connected to the lamp. According to responsiveness to incident light, AF emitted by the tissue may be classified as “retained” (RF), gained (GF), and reduced or lost (LF), in addition to two further conditions, i.e., porphyrinic fluorescence (PF) and diascopic fluorescence (DF) (Table 1) [45].

**Figure 2:**
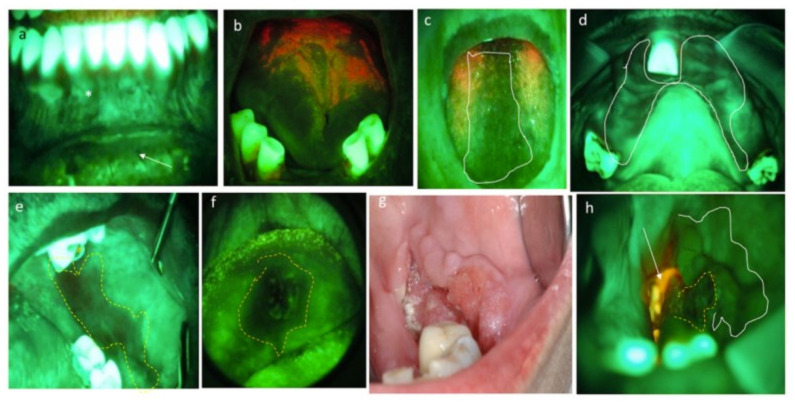
(**a**) RF at healthy mucosa: brighter at the gingiva (masticatory mucosa) than lip mucosa (covering mucosa). (**b**) PF, in red, due to the presence of bacterial plaque. (**c**,**d**) Reduced fluorescence due to inflammation. In these cases, anamnesis and lab tests completed the diagnosis of oral candidiasis. (**e**) Reduced fluorescence in a dysplasia of the cheek. (**f**) LF in an OSCC of the lip. (**g**,**h**) Clinical and AF pictures of an OSCC of the cheek. Inhomogeneous response to AF reveals LF at the central area of cancer (yellow dotted circle), pale reduction in fluorescence at its boundaries (white line), and PF due to necrotic tissue covered by bacterial plaque (white arrow).

RF is characteristic of the healthy oral mucosa, which emits an emerald green light, brighter at the masticatory mucosa than at the covering one (Figure 2a). Hyperkeratotic tissues emit a pale whitish-green light, due to the major content of keratin. The red/orange fluorescence is caused by porphyrins produced by micro-organisms colonizing the dorsal tongue and in the dental plaque (Figure 2b), and diascopic fluorescence is found in vascular lesions and lesions with a liquid-blood content. In the latter cases, the tissue initially appears dark; however, under ischemicization by pressure, it returns to normal fluorescence (blanching). Atrophic and inflamed mucosae emit reduced fluorescence due, respectively, to the lower keratin content and to the presence of blood that absorbs more light (Figure 2c,d). Lastly, proceeding from mild dysplasia (Figure 2e) to carcinoma (Figure 2f–h), the fluorescence progressively decreases, and even disappears in the case of neoplastic lesions, which, like a black hole, absorb all the incident light, thus appearing completely dark. Furthermore, neoplastic lesions can have an inhomogeneous response to fluorescence, as well as areas of loss of fluorescence alternating with normal fluorescent or hyperfluorescent areas due to superficial keratinization, as well as red areas due to the presence of necrotic material and bacterial plaque (Figure 2h).

Like TB, AF is also capable of highlighting occult early tumors and is a valid adjunct to presurgery identification of margins of excision of dysplastic epithelium/carcinoma escaping the naked eye. Furthermore, in a full-mouth AF, it also helps to screen subjects at risk and allows the follow-up of those with a history of OSCC or other SCC of the upper air/digestive ways.

Lane et al. found that “using histology as the gold standard, the device (AF) achieved a sensitivity of 98% and specificity of 100% when discriminating normal lesions from high-risk oral premalignant lesions and invasive SCC [44]”; nevertheless, we must consider the extreme operator-related variability in the usage and the highly subjective interpretation of the LF, which has to be contextualized with clinical suspects and anamnestic and lab tests, to exclude nontumoral causes of LF (mainly inflammations, exogenous and endogenous pigmentations, and atrophy of the mucosa inspected). For these reasons, AF and TB are both sensitive but not specific to OSCC and dysplasia diagnosis [38]. To overcome these limitations, the future improvement of AF could require digital image processing systems to objectivize and quantify the LF in a more precise and reproducible way [46,47].

## 4. Narrow-Band Imaging

Narrow-band imaging (NBI) is also known as virtual chromoendoscopy with magnification (VCM) and is extensively used in the diagnostic and follow-up management of pharyngeal and esophageal cancer, to distinguish tumor vascular patterns from other non-neoplastic affections [48].

NBI consists of the association of common endoscopes with magnification and conventional white-light source potentiated with narrow-bandwidth filters able to sequentially emit green-blue light, changing the spectral characteristics of the incident light [49]. Because hemoglobin strongly adsorbs green and blue light, and on the basis of the fact that shorter wavelengths lead to less light penetrating through the tissues and vice versa, due to the absorption and scattering process that occurs in the structures of the tissue, good contrast for the mucosa microvasculature of mucosa is obtained by the use of blue light, at the wavelength of 415 nm, which allows highlighting the more superficial vessels of the submucosa, and the use of green light, at the wavelength of 540 nm, which allows penetrating deeper into the tissue and can improve the visualization of the deeper vessels beyond the mucosa [50].

**Figure 3:**
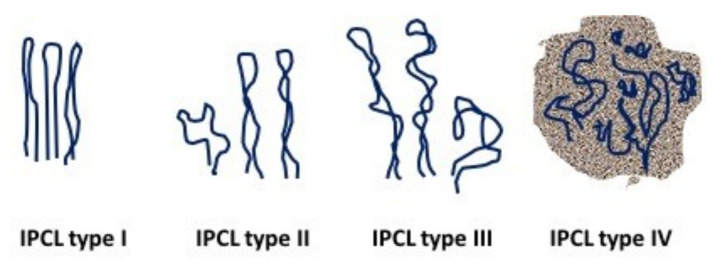
Oral IPCL classification according to Takano et al. [52] (Figure designed by Maria Contaldo).

The vessels analyzed by NBI are the so-called intraepithelial papillary capillary loops (IPCLs), which, close to the connective papillae of the oral mucosa, have an architecture characteristic related to the physiological or altered state of the overlying mucosa [51]. In 2010, Takano et al. [52] classified the IPCL patterns of oral mucosa into four types (Figure 3), corresponding to their progressing cancer-related disarrangements:

- Type I, regular loops;
- Type II, dilatated and crossing loops;
- Type III, elongated and meandering loops;
- Type IV, disrupted loops and neoangiogenesis.

The main works related to NBI in oral pathology (Table 2) have defined this method as a promising technique capable of discriminating the nature of a lesion according to its submucosal vascular pattern, with IPCL type IV predominantly found in OSCCs and high-grade dysplasia, associated with TNM advanced stages and histological grade of tumors. Conversely, IPCL types I and II are more frequently found in benign noninflamed lesions, in nondysplastic leucoplakia and other nonmalignant/nondysplastic oral lesions.

Regarding IPCL type III, its findings have been ubiquitously reported in leucoplakia with high-grade dysplasia, OSCC, and chronic inflammatory diseases and other benign lesions. Conditions that limit the use of NBI are as follows: markedly thick hyperkeratotic lesions, which obscure the vessel imaging, and lesions with hemorrhagic components, in which the blood impedes the visualization of the subepithelial capillaries. In these cases, NBI must be performed at the boundaries of the lesions, but the result can underestimate its malignancy [53].

Takano’s IPCL classification has been widely adopted in almost all studies on oral NBI, but Yang et al. proposed a simplified three-class classification, where IPCL patterns are equally statistically correlated with TNM, depth of tumor, and histological grade, as follows [55]:

- TD pattern: tortuous and dilated IPCLs;
- TE pattern: tortuous and elongated IPCLs;
- AD pattern: angiogenesis and IPCL destruction.

**Figure 4:**
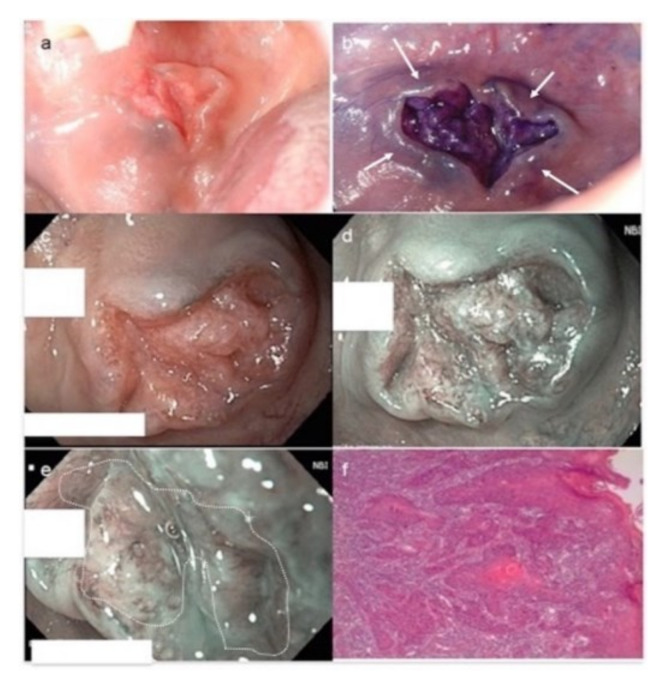
OSCC moderately differentiated. (**a**) Clinical photograph of a fissured exophytic lesion of the cheek. (**b**) Positivity to royal blue staining after TB test. Note bluish areas at the periphery of the ulceration (white arrows), expressing the persistence of suspected cells still retaining TB around the neoformation. (**c**) NBI imaging in white-light mode. At 60× magnification, NBI in white-light mode allows appreciating the epithelial covering of the fissured lesion. (**d**) At the same magnification, under narrow-band blue-green filters, NBI potentiates the capacity to appreciate subepithelial vessels. (**e**) At increased magnification (90×), the vascular features are more representatively seen: IPCL type IV, dilated and irregular in a brownish contest (white dotted circle), suggestive of cancer. (**f**) After surgery, conventional hematoxylin and eosin confirmed the diagnosis of OSCC G2, with infiltrative cancer cells and wide tumoral disarrangement of the tissue (original magnification 80×). In this case, TB and NBI help in the clinical setting before biopsy to orient the kind of surgical approach and to foresee the nature of the lesion, thus excluding traumatic injuries or non-neoplastic neoformations.

The NBI with magnification procedures usually follows the conventional white-light examination and it can orient the surgical margin of resection or the noninvasive follow-up of subjects with multiple lesions, as well as a history of OSCC and OPMDs, as the stepwise progression of different IPCL patterns correlates well with disease severity from normal oral mucosa to OSCC. In lesions showing more than one type of IPCL, the most advanced type detected is considered for classification. The use of NBI alone or in association with other diagnostic tools can potentiate the diagnostic process, as well as in cases of early cancers and high-grade dysplasia (Figure 4).

## 5. Optical Coherence Tomography

Optical coherence tomography (OCT) represents a valid tool in generating images of the tissue layers, by measuring the intensity of the backscattered light [60,61]. OCT guarantees a high axial and lateral resolution estimated, respectively, at 13–17 and 17–22 µm and a depth of penetration between 1 and 2 mm depending on the tissues examined [62,63]. OCT is based on low-coherence interferometry; a ray of light (an electromagnetic wave) reflects and diffuses on the tissues in different ways, which result in a delay time of the echo of reflected or backscattered light.

OCT stands as a useful and widely used device in various medical applications, and it has also been applied in oral science branches, proving to be an effective aid both on hard tissues and on mucous membranes; with a depth of penetration into tissues of 1–2 mm, OCT is considered suitable for oral mucosa [64], due to the thin thickness, between 0.2 and 1 mm. Numerous studies have shown that OCT can evaluate the macroscopic characteristics of epithelial, subepithelial, and basement membrane structures [64,65], with resolutions close to those of microscopy [66].

In 2008, Tsai et al. [65] developed an OCT comparison of healthy mucosa and mucosa affected by malignant and premalignant lesions. Different samples were subdivided into four groups: healthy mucosa (control group), epithelial hyperplasia, moderate dysplasia, and squamous cell carcinoma of the oral cavity (OSCC). The results obtained with histology were then combined with OCT data, reporting the analysis and statistical results of OCT images, using three diagnostic indicators: standard deviation (SD) of a scan signal profile, the exponential decay constant (α) of a scan spatial frequency spectrum, and epithelial thickness (T). In the oral mucosa of the three study groups, data obtained showed that the standard deviation increases, the α parameter decreases, and the epithelium becomes thicker. SD and α are satisfactory diagnostic markers for moderate dysplasia and neoplastic lesions. On the other hand, the T parameter comes out as a good diagnostic indicator for hyperplasia and moderate dysplasia, especially when compared with the epithelial thickness of healthy mucosal areas of the same patient.

Wilder-Smith et al. [64] performed an in vivo OCT analysis of 50 oral leukoplakia (OL) and erythroplakia (OE); among these, a dysplastic lesion showed epithelial thickening with loss of stratification in the epithelial layers, deeper than the healthy oral mucosa comparison. An oral squamous cell carcinoma (OSCC) was depicted in the OCT images; thus, detailed features were highlighted: variability of the epithelial thickness, as well as areas of erosion and invasion in the subepithelial layers, with loss of integrity of the basal membrane. Each lesion was subjected to biopsy and histological examination. Two different blinded operators performed the diagnosis on each lesion, first on the basis of OCT scanning and then on the histological examination, achieving a high correspondence between the two diagnoses.

A recent in vitro study by Jerjes et al. [66] compared the epithelial thickness OCT estimation with the OSCC biopsy tissue samples ex vivo analyses; changes in epithelial tissue are related, in fact, to the increase in the turnover rate of premalignant and malignant lesions [65,66,67]. Measurements performed with OCT were found to be superimposable with those on a histological sample. Epithelial thickness, therefore, can help improve the identification and diagnosis of suspicious lesions, especially in combination with the assessment of tissue architectural changes.

Optical coherence tomography devices have proven the feasibility of assessing the oral mucosa, and the employment of this technique could contribute to the early diagnosis of oral squamous carcinoma. OCT images provide information on the epithelium, on the basal membrane and the lamina propria, enabling reliable measurement of epithelial thickness in vivo and ex vivo [65,66,68]. Several variables, such as epithelial thickness and basal membrane integrity, have turned out identifiable by OCT, contributing to the obtention of information on the malignant nature of the oral lesions. Nevertheless, the OCT image quality remains operator-sensitive [60]; furthermore, the rigid device’s probe makes it difficult to examine the less accessible areas of the oral cavity [68,69].

Biopsy and histological examination are still considered the gold standard for evaluating lesions of the oral cavity, but OCT can represent an effective noninvasive in vivo examination, useful for the early diagnosis of preneoplastic and neoplastic lesions.

## 6. High-Frequency Ultrasound (US)

Ultrasonography (US) is a diagnostic procedure that uses sound waves, emitted by piezoelectric crystals, and their echoes are capable of producing images of anatomical structures [70]. Ultrasound is routinely used in various branches of medicine, such as gynecology, gastroenterology, cardiology, and angiology; with regard to the head and neck region, it is used to detect structural abnormalities and/or tumors of the salivary glands [71,72]. The use of this technique in the oral cavity is less frequent but, in any case, different uses have been reported in the literature: from the study of periodontal and peri-implant tissues to the clinical and surgical characterization of benign and malignant lesions of the oral mucosa [73,74,75].

**Figure 5:**
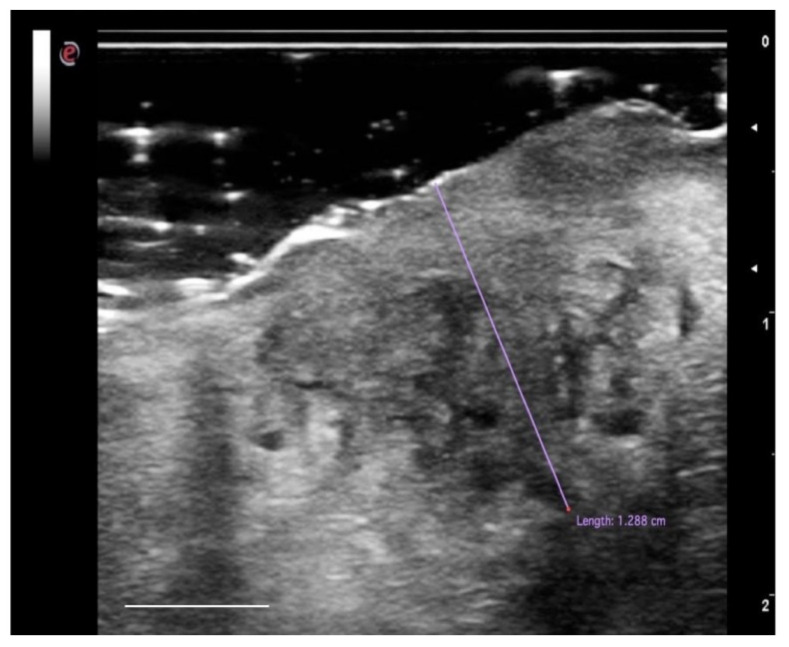
High-definition US imaging (frequency 18 MHz) of a suspicious lesion of the tongue margin. The high-definition ultrasound image shows a hypoechoic area with a thickness of 1.288 cm (TT), highlighted with the purple line, in the context of the lingual parenchyma (which does not show alterations in echogenicity). The histopathological analysis revealed the presence of OSCC of the lingual margin. Scale bar, 0.5 cm.

Intraoral ultrasound examination has the advantage of having direct contact with the region of interest, maintaining the characteristics of noninvasiveness, rapidity in execution, and repeatability, increasing the methods of possible applications [76]. Ultrasound techniques are considered particularly useful in determining the interface between tumor tissue and the surrounding myo-architecture [77,78]. A significant correlation has been reported between the histological aspects of the sample and the ultrasound image. OSCC appears as a rather defined hypoechoic lesion [76,79]. In other works [80,81], OSCC lesions were reported as hypoechoic with irregular margins and, in most cases, not well defined (Figure 5). Natori et al. reported that tumors with more invasive characteristics may show irregular and unclear margins on the ultrasound image [78].

The first intraoral application of ultrasound for OSCC imaging was initially described by Shintani et al. in 1997. This study aimed to evaluate and measure the extent of tumor thickness (TT), comparing it with the pathological sections and reporting a good agreement between the two techniques (*R* = 0.985). In a subsequent study [76], Shintani and colleagues compared the measurements of TT obtained with the ultrasound examination not only with the histology but also with computed tomography (CT) and magnetic resonance imaging (MRI). This study showed that intraoral ultrasound was more accurate in detecting and measuring tumors of the oral mucosa (*R* = 0.988), compared to CT (*R* = 0.976) and MRI (*R* = 0.911). Other studies [82] confirmed the capabilities of the US imaging technique in detecting lesions smaller than 1 mm thick (with a very high agreement with the histological examination), or at least lesions of about 1 mm of thickness were detectable in the intraoral ultrasound image in the context of the tongue parenchyma [78]. In this way [83], the ultrasound examination proved to be more accurate than MRI in measuring the TT. Accuracy increases in T1 and T2 tumors; for advanced-stage tumors (classification T3-T4), the determination of the thickness of the tumor in ultrasonography will be less accurate.

However, as the TT considers the entire extent of the tumor from the surface to the point of deepest invasion, this measure may underestimate the potential invasiveness in ulcerated forms (mainly in the tongue and floor of the mouth SCC) and overestimate the aggressiveness of the exophytic neoplasia limited within the epithelial layers. For these reasons, in 2018, the American Joint Committee on Cancer Union for International Cancer Control (AJCC-UICC) introduced another feature to be considered in the process of OSCC tumor staging: the depth of invasion (DOI) of the tumor [84]. DOI considers the subepithelial extent of the carcinoma because it measures, in millimeters, the distance from the basement membrane of the epithelial surface to the deepest point of tumor invasion and has proven to be a more reliable prognostic factor than the thickness of OSCC for predicting regional node involvement and the survival of patients with OSCC [85]. Despite both TT and DOI being routinely histologically measured after surgical procedures, the recent literature has reported the possibility of evaluating the DOI preoperatively, through high-frequency US, with a sensitivity and specificity of 91–93.1% and 100%, respectively, to assess the infiltration of the tumor beyond the lamina propria into the submucosa compared with histological measurements [73,86].

Several studies have proposed the use of this instrument as an aid to surgical resection to mark the deep tumor margin [87]. Other authors [88] proposed the intraoperative use of the ultrasound probe, to guide the resection of the deep margins with the use of eco-reflective instruments.

In conclusion, US imaging is an in vivo, noninvasive, and real-time diagnostic method that allows an accurate measurement of the tissues of the oral mucosa, and it can also be useful as a surgical aid. The value of this imaging technique appears to be superior, in terms of dimensional measurement, to techniques such as CT and MRI, in lesions with a thickness ≤5 mm. However, this instrument is operator-dependent and the learning curve is still very high.

## 7. In Vivo Confocal Microscopy

While the technologies reported above give additional but partial information about some neoplastic/dysplastic signs of a lesion, in vivo confocal microscopy (CM) goes further and allows performing a proper virtual biopsy of the tissues in their living context, offering real-time cytological and histological details, without anesthesia, surgery, or adverse reactions.

In vivo confocal microscopes use a nonharmful incident laser light at specific wavelengths to stimulate the emission of fluorescent or refracted light from the living tissues, according to the presence of fluorophores or proportionally to the refractive indices of the various compounds of the tissue, respectively. In fluorescent confocal microscopy (FCM), an exogenous fluorescent substance is administered to the tissue and, due to its accumulation in different tissue components based on its chemical properties, it can highlight cellular and subcellular features by returning fluorescent light from the illuminated focal plane [89]. In reflectance confocal microscopy (RCM), the system detects the refracted light emitted specifically by the various cellular compounds on the basis of their refractive index [90]. In both systems, the light emitted from the tissues is collected point by point by a detector, the signals are converted into grayscale pixels, and an integrated software then rebuilds the en face optical section at microscopic resolution, in a grayscale frame visible on a monitor [91].

While FCM has been mainly reported in ex vivo studies [92] with few exceptions [93,94], florid literature on the in vivo RCM has existed over 30 years, and this technology has offered continuous advances in the field of noninvasive real-time microscopic imaging of the living tissues.

Used for the first time in ophthalmology [95], RCM mainly spread to dermatology in the last 30 years, for imaging melanocytic and epithelial malignancies, interface dermatitis, and other skin diseases [96,97,98,99], while, more recently, it has also begun to be applied in dentistry and oral pathology, for imaging both hard [100,101,102,103] and soft tissues [104,105] of the oral cavity.

After pilot studies to validate the method [93,106,107], research has focused on highlighting the peculiarities of various pathologies affecting the oral mucosa, such as oral lichen planus and other inflammatory oral diseases [108,109,110,111,112], pigmented lesions of the lips [113,114,115,116], and actinic cheilitis [117,118], as well as to define some “confocal features” peculiar of preneoplastic and neoplastic oral lesions [119,120,121]. The RCM indicators of OSCC overlap the conventional histologic criteria, except the classical OIN grading [122,123], which, being performed on transversal sections of the tissue, is not allowed by the horizontal virtual slicing obtained by in vivo RCM. However, it is sufficient to change the point of view and refer, for the future, to novel and peculiar RCM indicators for a novel grading of cancers.

To date, the main in vivo RCM findings reported in OSCC are as follows: pleomorphisms of keratinocytes, necrotic keratinocytes, nuclear ploidies (indirectly defined by the presence of multiple and/or rod nucleoli within the cells), increased nuclear/cytoplasmic ratio, abnormalities in size and shape of subepithelial blood vessels, monstrous connective tissue papillae obscured by inflammatory infiltrates, keratinocytes visibly escaping to submucosal layers, and inflammatory cells infiltrating the epithelium. Furthermore, in vivo RCM can show the completely disarranged architecture of the various epithelial and subepithelial layers; the classical regular frosted-glass pattern of the spinous layer appears subverted, keratin pearls are visible in well-differentiated OSCCs, crowded keratinocytes cap the connective tissue papillae, and higher amounts than normal of the epithelial–connective tissue interdigitations are represented by an increase in the density of the connective tissue papillae [120].

Due to the lack of stratum corneum and stratum lucidum present in the skin, oral mucosa can be easily imaged up to the submucosal layers, except for the thick hyperkeratinized lesions, whose strong superficial keratin content, highly refractive, obscures the light penetration to the lower layers.

**Figure 6:**
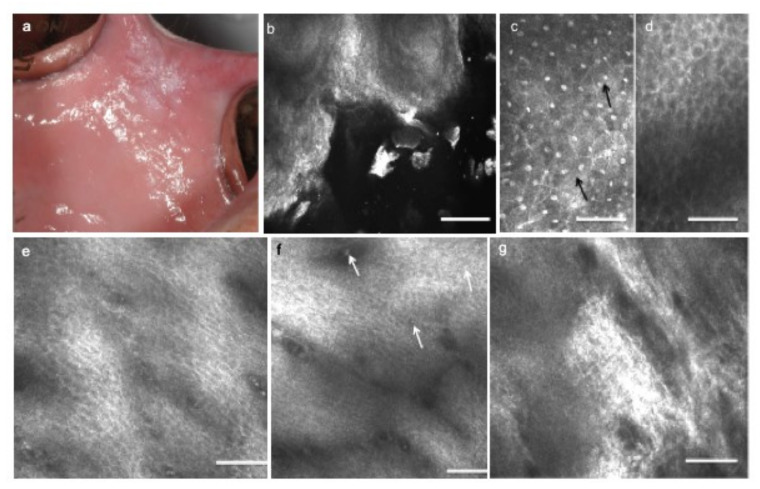
Leucoplakia of the cheek with dysplasia. (**a**) Clinical presentation of leucoplakia of the left cheek in a 41 year old male, smoker. (**b**) On RCM imaging, the superficial keratinization is expressed by bright areas with no identifiable cells alternated with brighter corneocyte aggregates. (**c**) At the upper spinous layer, polymorphic keratinocytes different in size are quite irregularly arranged and some of them show more than one nucleolus (black arrows). (**d**) At the lower spinous layer, the architectural pattern, slightly irregular, is intermediate between frosted glass and honeycombed, with thickened intercellular spaces. (**e**) Going deeper, the shadow of the connective tissue and the inflammatory exudate become visible as irregular dark areas. (**f**) Inflammatory cells, identified as small roundish dots, can be found in the epithelium and surrounded by dark hazy areas due to inflammatory infiltrates (white arrows). Connective tissue papillae lose their regular small roundish shape and become confluent in large elongated papillae. (**g**) At the submucosal layers, a strongly bright connective tissue expresses a chronic reaction, and connective bundles are irregularly arranged. Scale bars, 100 mm.

The improvement of ergonomics of the actual commercially available devices or the production of oral-dedicated ones will help the widespread use of RCM in clinical practice for experimental studies on follow-up of OPMDs and the responsiveness to therapies, without the need for recurrent biopsies, as well as guide surgeons to the margins free from cancer and shorten the time for OSCC treatment (Figure 6).

## 8. Discussion

OSCC is a malignancy associated with high mortality in its advanced stages. Despite easy access to the oral cavity, the multiform clinical presentations of early OSCCs are not always suggestive of cancer, may mimic other oral lesions, and, when misdiagnosed or mistreated, worsen and acquire invasiveness and metastasis. OPMDs may underline cancerization not identifiable with the routine conventional examination, thus requiring biopsy and histopathological assessment. Large evident OSCCs must be preliminarily mapped to orient surgeons toward the most representative tumoral area for choosing the more appropriate therapeutic approach.

The diagnostic procedure of these conditions requires time, and, in lesions requiring prolonged follow-up over time, more biopsies are necessary. Adjunctive noninvasive imaging techniques performable at the chairside and that offer additional information in real time without hurting the patient could significantly improve the quality and the speed of the diagnostic procedure, enable the patient’s compliance, and allow discovering occult OSCCs and OPMDs cancerization. Each device has its qualities and limitations; some focus on two-dimensional macroscopic details, while others allow the measurement of the thicknesses and the depth of the lesions. RCM also allows the real-time preliminary consideration of the cytological and histological characteristics (Table 3).

Some of these techniques are conventionally used in clinical practice, such as vital (TB/LI) and virtual (AF) staining. Despite offering additional information on the nature and the extent of the lesions, their major limitations are the two-dimensional appraisal of the lesions and the operator-related interpretation. To overcome this second limitation, some authors have proposed digital image processing, using an artificial neural network [50,51] or fractal analysis of images [124] to objectivize the interpretation.

A variant of AF is fluorescence induced by exogenous fluorophores, such as toluidine blue or 5-amino-laevulinic acid [125]. In these cases, exogenous fluorophores are not only used for diagnostic purposes but also for low-dose laser therapy techniques such as photodynamic therapy (PDT), which is a minimally invasive treatment for malignant and premalignant lesions, previously diagnosed by conventional biopsy, according to the selective destruction of only cells that retain fluorophores topically administered to the dysplastic/neoplastic lesion [126,127,128,129,130,131].

Recently, the off-label use of AF to examine the bacterial-induced porphyrin fluorescence on the tongue has allowed highlighting the negative association between porphyrin fluorescence and *Candida* colonization of the oral mucosa. Despite very preliminary, these trials open a new scenario for the detection of oral fungal infections and could offer a different, cost-effective, and real-time approach to the detection of oral mycosis [132].

Despite the techniques above, HF US and OCT allow us to consider the three-dimensionality of the tumoral mass and accurately measure its thickness and the depth of infiltration.

Furthermore, NBI adds relevant information about the vascular pattern, with IPCL type 4 being strongly associated with OSCC and high-grade dysplasia. RCM allows the optical biopsy of the tissue, allowing in vivo and real-time identification of the presence of OSCC microscopic indicators.

However, despite its undoubted support in providing optical biopsy of living tissues with a resolution close to conventional histology, the widespread use as a chairside diagnostic method in oro-dental clinical practice of in vivo CM is still limited by some important restrictions. These are mainly the lack of medical devices ergonomically adaptable to each site of the oral cavity, and the need for experience and high-level training to correctly interpret the cytological and histological findings of CM. However, CM is extraordinarily useful in the clinical practice to also orient diagnosis of non-neoplastic diseases, as an alternative to conventional histology in some selected cases and for research purposes, to correlate molecular and classical histological findings within CM features [133,134,135,136], and we hope that technical advances can soon be achieved.

## 9. Conclusions

In conclusion, despite the described techniques not always being able to replace conventional histology, if used properly and with the right know-how, they are a resource and to improve the quality of the diagnostic path in oral pathology and dentistry in the future, thus allowing the conduction of noninvasive follow-up of subjects at risk for OSCC or with history of SCC of the upper air/digestive ways, to discover signs of malignancies in OPMDs, and, in the case of advanced, large, multifocal OSCC, to presurgically map its superficial extent and its DOI, to orient surgeons to better approach the OSCC for a conclusive diagnosis and therapeutic excision in free margins.

**Figure 7:**
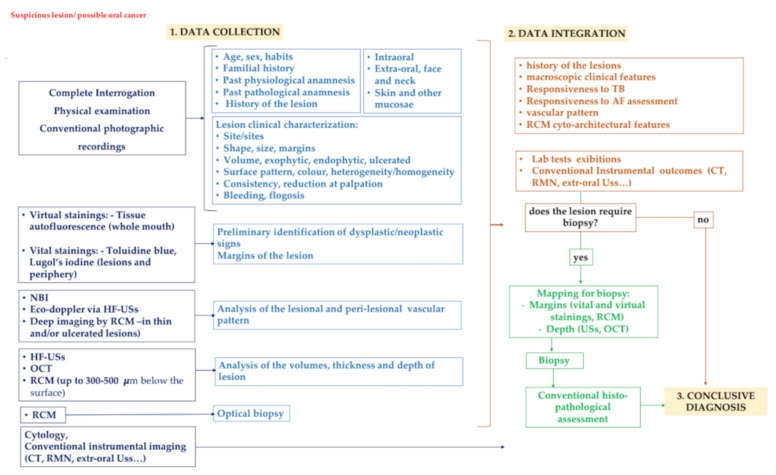
The proposed three-step diagnostic algorithm in oral pathology considering three consecutive phases: 1, data collection; 2, data integration; 3, conclusive diagnosis. (1) During the first phase, a series of noninvasive imaging techniques are performed to identify some additional diagnostic markers in addition to those obtained with conventional clinical examination and collection of anamnestic data. (2) In the second step, all data are integrated to establish the need for diagnostic biopsy. (3) In some cases, the clinical and instrumental characteristics point directly toward a conclusive diagnosis. On the other hand, when a biopsy is required, the lesion is reconsidered, with the noninvasive techniques considered, to map the lesion and suggest the correct margins and depth to the surgeons; then, the specimen is subjected to conventional histopathological evaluation and the conclusive diagnosis is obtained.

With this in mind, considering the potential of each method, we propose an idealistic three-step procedure (Figure 7) that simplifies, improves, speeds up, and implements the diagnostic process. A three-step diagnostic pathway that considers all of these techniques could reduce the need for exploratory biopsies and lead to the timely and more precise eradication of OSCC.

In the proposed three-step diagnostic protocol, a series of noninvasive imaging techniques are introduced to improve the presurgical diagnostic accuracy of suspicious or frankly tumoral oral lesions. They are placed, chronologically and conceptually, before the conventional bioptic procedure.

In the first phase (data collection), in addition to the diagnostic indicators obtained with conventional clinical examination and collection of anamnestic data, a series of adjunctive investigations allow obtaining additional information on the suspected lesion or carcinoma, in the context of the classical clinical–anamnestic protocol. The vital stains and the autofluorescence allow dichotomizing the diagnostic suspect as positive/negative. NBI and doppler ultrasound via HF US provide details on the vascular pattern and possible tumor neoangiogenesis. A preliminary detailed measurement of the tumor size (superficial margin extent, TT and DOI) is obtained with HF US and OCT. Lastly, the optical biopsy allowed by in vivo RCM offers real-time cytoarchitectural information of the lesion at microscopic resolution.

Having some or all these methods ideally available at the chairside, the performance of the examinations can be contextually planned during the first clinical evaluation or deferred for mere organizational reasons. In any case, the results can be integrated with conventional instrumental investigations and lab tests.

The second step (data integration) comes before the biopsy (when required); after a possible re-evaluation in case of doubts or clinical changes, all the information collected allows the surgeon to define the need for surgery, plan the type of approach (incisional vs. excisional biopsy), and accurately define the three-dimensional extension of the incision/enucleation, to be able to conclude the diagnostic procedure with the conventional histopathological evaluation of the specimen (conclusive diagnosis).

In conclusion, the proposed empowered diagnostic protocol allows the thorough presurgical definition of the suspicious lesion and its features, the mapping of extended or multifocal lesions to define the elective sites for biopsy, and the discrimination of lesions with different prognostic significance and surgical/therapeutic approach. This methodology also allows monitoring more conservatively those lesions requiring a “wait and see” approach, and it can be advantageous for following up the evolution of chronic lesions and diseases, thus reducing multiple biopsies, when unnecessary. Furthermore, it can be assumed that the patient will be more compliant and motivated to respect the periodic follow-up.

Confronting OSCC and saving the patient is a battle against time; while some of these technologies are still under study and require practice and experience, we look forward to the near future where they will be applied clinically and routinely to increase patient survival and improve quality of life after cancer.

## Figures and Tables

**Table 1:** AF emitted according to clinical conditions. Each part of the figure on the right, correspond to the AF output according to the condition listed on its left on the same line.

Gained fluorescence	Linea alba Fibroma Hyperkeratotic lesions	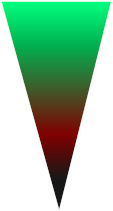
Retained fluorescence	Healthy mucosa	
Diascopic fluorescence	Vascular lesions Mucocele	
Porphyrinic fluorescence	Bacterial plaque	
Reduced fluorescence/ Loss of fluorescence	Atrophic lesions Inflammatory lesions Ulcerated lesions Pigmented lesions Dysplasia Carcinoma	

**Table 2:** NBI in oral malignant and nonmalignant lesions.

First Author, Year, Country	Type of Study (Lesions Considered)	Types of Oral Lesions	*N* of Patients Enrolled (with Cancer)	Mean Age (Range) year	Sex M:F	Main Findings				Other Findings/Conclusions
						IPCL I	IPCL II	IPCL III	IPCL IV	
Takano et al. 2010, Japan [52]	Case/control	Neoplastic/non-neoplastic oral lesions	41	52.34 (23–83)	26:15	10 HM	2 aphthae, 1 leucoplakia	4 leucoplakia, 12 OSCC	11 OSCC	IPCL types associated but NS with TNM, histological grading, and smoking
Yang et al. 2012 Taiwan [53]	Cohort	Oral leucoplakia	154	53	130:24	50 leucoplakia without dysplasia; 10 leucoplakia with dysplasia	48 leucoplakia without dysplasia; 4 leucoplakia with dysplasia	28 leucoplakia with dysplasia; 5 leucoplakia with OSCC	9 leucoplakia with OSCC	IPCL type IV was specifically present only in cases with OSCC
Yang et al. 2012 Taiwan [54]	Cohort	Clinically homogeneous oral leucoplakia)	160	51 (22–75)	135:25	35 thin leucoplakia without dysplasia; 94 thick leucoplakia (68 nondysplastic, 24 LGD, 2 IGD)	29 thick leucoplakia (6 nondysplastic, 20 LGD, 3 IGD)	2 thick leucoplakia (1 HGD, 1 invasive OSCC)	0	Authors strongly recommend biopsy if IPCL type II, III, or IV is found by NBI
Yang et al. 2013, Taiwan * [55]	Cohort	OSCC	80	54 (33–85)	73:7	13, TD pattern (tortuous and dilated IPCLs)	31, TE pattern (twisted and elongated IPCLs)		35, AD pattern (angiogenesis and destruction of IPCLs)	IPCL patterns statistically correlated with TNM, depth of tumor, and tumor histological grade
Shibahara et al. 2014, Japan [56]	Cohort	Neoplastic/non-neoplastic oral lesions	121	61	-	IPCL type 1–2 77 oral healthy mucosa and non-neoplastic oral diseases; 4 malignant tumors		IPCL type 3–4 8 oral healthy mucosa and non-neoplastic oral diseases; 48 malignant tumors		Advanced IPCLs associated with OSCC
Yang et al. 2015, Taiwan [57]	Cohort	Oral erythroplakia	72	55	66:6	6 squamous hyperplasia	5 squamous hyperplasia; 18 LGD; 11 IGD; 4 HGD	1 squamous hyperplasia, 4 LGD; 6 IGD; 13 HGD; 1 OSCC	3 OSCC	IPCL type 4 is an indicator of HGD and OSCC
Contaldo et al. 2017, Italy [58]	Observational	Neoplastic/non-neoplastic oral lesions	31	60 (23–88)	16:15	3 HM; 1 exfoliative cheilitis; 1 PV; 1 scrotal tongue; 2 hyperkeratosis; 1 PVL; 5 OLP, 1 nondysplastic chronic ulcer	3 OLP; 1 hyperplastic Candidiasis; 1 LGD; 1 PV	1 geographic tongue; 2 OLP; 1 IGD	1 type IV hypersensitivity reaction; 1 IGD; 4 OSCC	Lack of OSCC false negative to NBI
Guida et al. 2019, Italy [59]	Cohort	Neoplastic/non-neoplastic oral lesions	98	61	58:40	4 LGD; 3 OLP; 11 frictional keratosis	7 LGD; 14 OLP; 18 frictional keratosis; 1 OSCC	7 LGD; 13 OLP; 1 frictional keratosis; 1 OSCC; 1 PVL; 1 HGD	2 LGD; 13 OLP; 17 OSCC; 2 PVL; 3 HGD	OLP did not influence sensitivity and co-occurring cancer detection at NBI

Legend: HM, healthy mucosa; LGD, low-grade dysplasia; IGD, intermediate-grade dysplasia; HGD, high-grade dysplasia; OLP, oral lichen planus; PV, pemphigus vulgaris; PVL, proliferative verrucous leucoplakia; NS, not statistically significant. * In this work authors classified IPCLs in three patterns different from Takano.

**Table 3:** Main characteristics of the techniques considered.

	Vital/Virtual Staining	NBI	High-Frequency US	OCT	In Vivo CM
2D imaging	+	+	+	+	+
3D imaging	−	−	+	+	+ *
Macroscopic shape	+	+	+	+	−
Inflammation signs	+	+	+	−	+
Blood vessel pattern	−	+	−	−	+
ECJ boundaries	−	−	+	+	+
Cyto-histological features of epithelial strata	−	−	−	−	+
Cyto-histological features of subepithelial strata	−	−	−	−	−/+
Measures of parameters	−	−	+	+	+
Hyperkeratinized lesions	−	−	+	+	−/+
Early signs of cancers detection	+	−	−	−	+
Depth of invasion	−	−	+	+	−/+
Total score ^a^	3	4	8	7	11

Legend: ECJ, epithelial–connective junction; +, possible; −, not possible; −/+ not always possible. * 3D reconstructions are available for some RCM devices. ^a^ Total score refers to the maximum types of features appreciated in each technique.
